# Bi-directional encoding of context-based odors and behavioral states by the nucleus of the lateral olfactory tract

**DOI:** 10.1016/j.isci.2021.102381

**Published:** 2021-03-31

**Authors:** Yuta Tanisumi, Kazuki Shiotani, Junya Hirokawa, Yoshio Sakurai, Hiroyuki Manabe

**Affiliations:** 1 Laboratory of Neural Information, Graduate School of Brain Science, Doshisha University, Kyotanabe City, Kyoto 610-0394, Japan; 2 Research Fellow of the Japan Society for the Promotion of Science, Chiyoda Ward, 102-0083 Tokyo, Japan

**Keywords:** Neuroscience, Behavioral Neuroscience, Sensory Neuroscience

## Abstract

The nucleus of the lateral olfactory tract (NLOT) is not only a part of the olfactory cortex that receives olfactory sensory inputs but also a part of the cortical amygdala, which regulates motivational behaviors. To examine how neural activity of the NLOT is modulated by decision-making processes that occur during various states of learned goal-directed behaviors, we recorded NLOT spike activities of mice performing odor-guided go/no-go tasks to obtain a water reward. We observed that several NLOT neurons exhibited sharp go-cue excitation and persistent no-go-cue suppression responses triggered by an odor onset. The bidirectional cue encoding introduced NLOT population response dynamics and provided a high odor decoding accuracy before executing cue-odor-evoked behaviors. The go-cue responsive neurons were also activated in the reward drinking state, indicating context-based odor-outcome associations. These findings suggest that NLOT neurons play an important role in the translation from context-based odor information to appropriate behavior.

## Highlights

• We recorded NLOT spike activities in the odor-guided goal-directed behaviors
• NLOT neurons were classified into five response types in the odor-sampling epoch
• Many NLOT neurons exhibited go-cue excitation and no-go-cue suppression responses
• The bidirectional responsive neurons were also activated in the reward drinking

## Graphical abstract

**Figure:**
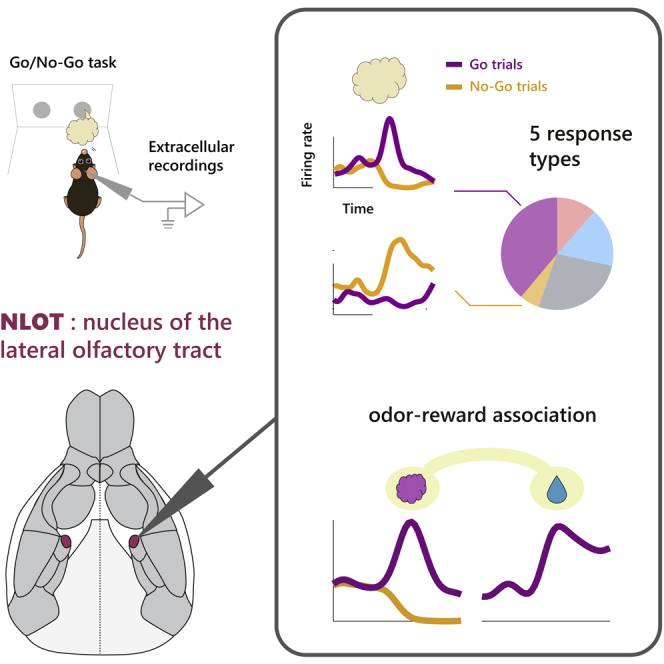

## Introduction

The nucleus of the lateral olfactory tract (NLOT) is part of the olfactory cortex that receives direct sensory inputs from the olfactory bulb and the olfactory cortex, including the piriform cortex (Luskin and Price, 1983; Price, 1973). Alternately, it also receives projections from the anterior amygdaloid area, anterior cortical and posterolateral cortical amygdaloid nuclei, and amygdalo-piriform transition area and forms part of the olfactory amygdala (Alheid et al., 1995). Some authors have considered the NLOT to be a component of the olfactory cortex (Price, 1973; Swanson and Petrovich, 1998), whereas others have regarded it as a component of the cortical amygdala areas that plays a critical role in generating odor-driven behaviors (de Olmos et al., 2004). The NLOT not only has a bidirectional connection with the olfactory bulb and piriform cortex but also strongly innervates the basolateral amygdala (BLA) and ventral striatum (Luskin and Price, 1983; Price, 1973; Santiago and Shammah-Lagnado, 2004). Owing to its anatomical features, it is possible that the NLOT is involved in odor-evoked motivational behaviors.

In addition to this anatomical evidence, a recent study (Vaz et al., 2017) has shown functional evidence that NLOT integrity is required for normal functioning of the olfactory system. Researchers have conducted a series of behavioral tests using rats with bilateral excitotoxic lesions of the NLOT. The NLOT-lesioned rats exhibited severe olfactory deficits with an inability to detect and discriminate between odors.

Despite the accumulation of knowledge, there are no reports of *in vivo* recording of neuronal activity in the NLOT. Therefore, the electrophysiological features of NLOT neurons on odor-evoked motivational behavior have not been clarified. The purpose of our study was to investigate how neural activity is modulated by motivational processes that occur during various behavioral states in a goal-directed task. Here, we recorded the neural spike activities in the NLOT of freely moving mice performing an odor-guided go/no-go task. We observed that the majority of NLOT neurons exhibited go-cue excitation and no-go-cue suppression responses triggered by an odor onset. The bidirectional cue encoding strongly contributed to the NLOT neuron population dynamics before executing cue-odor-evoked behaviors; additionally, the go-cue responsive neurons encoded a reward drinking state, indicating context-based odor-outcome associations. Our results suggest that the NLOT is critical for encoding context-based cue-outcome signals and may play an important role in the translation of odor stimulus information to appropriate behavior.

## Results

**Figure 1:**
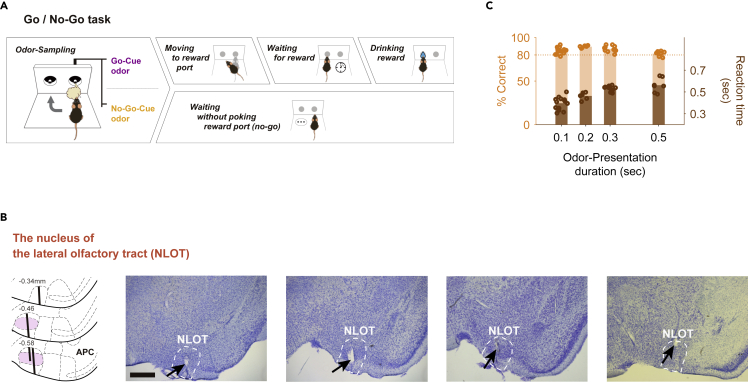
Odor-guided go/no-go task and nucleus of the lateral olfactory tract (NLOT) (A) Time course of the odor-guided go/no-go task. Behavioral epoch temporal progression from left to right. (B) Nissl-stained frontal section (arrows indicate tips of the tetrodes) and recording tracks (vertical thick lines) of the NLOT. The pink areas show layer II of the NLOT. APC, anterior piriform cortex. Scale bar, 500 μm. (C) The reaction times (from the onset of odor presentation to the timing of nose withdrawal, right axis, brown) in the odor-guided go/no-go tasks without keeping the mice's nose inserted into the odor port. To test the time lag between the start of odor stimulation and the arrival of the odor molecules to the mouse's nose, we verified odor presentation durations of 100, 200, 300, and 500 ms in each session. When the duration of odor presentation was shortened, the mice showed a shorter reaction; however, the behavioral accuracy remained >80% (left axis, orange), indicating that the arrival of the odors was within 400 ms after odor valve opening, and that the mice could make decisions within the period.

We obtained recordings from 365 well-isolated neurons in the NLOT (median baseline firing rate = 0.90 Hz, interquartile range = 0.23–3.07 Hz; median spike width = 0.53 ms; median inter-spike interval = 134 ms) of four mice performing odor-guided go/no-go tasks (Figures 1A and 1B). Briefly, the go trial required the mice to first sample a go-cue odor stimulus presented at an odor port and then to move to a reward port to obtain a water reward. Conversely, the no-go trial required the mice to first sample a no-go-cue odor stimulus presented at the odor port and then to stay near it to wait for the next trial. It is important to note that the mice were required to keep their nose inserted into the odor port during odor presentation (500 ms). After the mice were well trained, their behavioral accuracy remained >80% throughout the session. For all mice, the median of the odor-sampling epoch (the time from odor valve opening until the withdrawal of the snout by the mouse from the odor port) was 788 ms (interquartile range: 669–962 ms) in the go trials and 642 ms (interquartile range: 562–798 ms) in the no-go trials (44 sessions from four mice).

However, it is possible that these reaction times reflect the time lag between the start of odor stimulation and the arrival of the odor molecules to the mouse's nose. We, therefore, examined how quickly the mouse decides on its behavior by shortening the duration of odor presentation and measuring the reaction time (from the onset of odor presentation to the timing of nose withdrawal) (Figure 1C). When the duration of odor presentation was shortened without the forced nose-poking during odor presentations, the mice showed shorter reaction times; however, the behavioral accuracy remained >80%. These results indicate that the arrival of the odors was at least within 400 ms after the opening of the odor valve, and the mice could make decisions within the odor presentation. In the following sections, we describe our analyses of the neural activity recorded during odor-sampling and the following odor-guided behaviors.

### Five-type classification of NLOT neurons based on odor-sampling epoch response

**Figure 2:**
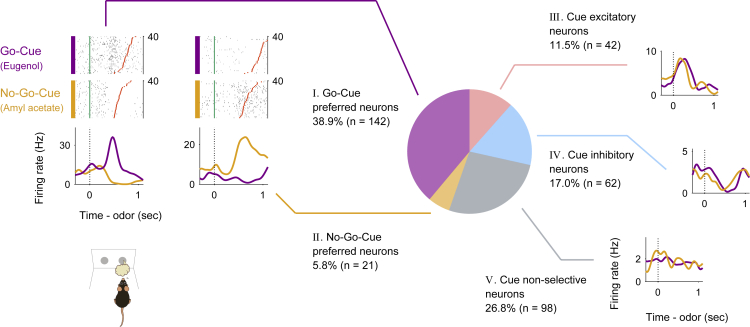
Nucleus of the lateral olfactory tract (NLOT) neuron activity patterns during the odor-guided go/no-go task Example firing patterns of NLOT neurons during the odor sampling epoch (the time from odor valve opening to odor port exit) in the odor-guided go/no-go task. Each row contains the spikes (black ticks) for one trial, aligned to the time of odor valve opening (corresponding to the odor port entry, green ticks). Red ticks refer to the times of odor port exit. The correct trials are grouped by odor, and within each group, the trials are sorted by the duration of the odor-sampling epoch (40 trials selected from the end of the session are shown per category). Histograms are averaged across odors and calculated using a 20 ms bin width and smoothed by convolving spike trains with a 60-ms-wide Gaussian filter (purple, go-cue odor; orange, no-go-cue odor). The vertical dashed lines indicate the time of odor valve opening. NLOT neurons were classified into five types (purple pie, type I; orange pie, type Ⅱ; pink pie, type Ⅲ; light blue pie, type Ⅳ; and gray pie, type Ⅴ) based on the odor-sampling epoch response (Figure S1).

As the NLOT receives direct inputs from the mitral cells of the olfactory bulb, we first focused on the neural activity during the odor-sampling epoch. We observed that the firing rates of the NLOT neurons increased or decreased during the odor-sampling epoch. For a large subset of neurons, these firing rate changes depended on whether the presented odor was a go-cue or no-go-cue (examples shown in Figure 2, left). To quantify the dependence of the firing rate on cue odor presentation, we used a receiver operating characteristic (ROC) analysis approach. We calculated the firing rate changes from baseline (1,000−0 ms before the end of the inter-trial interval) during the odor-sampling epoch. Across the population, 73.2% of the NLOT neurons exhibited significant responses to at least one cue odor presentation (Figure S1, p < 0.01, permutation test). In this cue odor selective population, we also calculated the preference for go-cue and no-go-cue odor presentation. We observed that 53.2% of the population showed a significant go-cue odor preference, whereas 7.9% showed a significant no-go-cue odor preference (Figure S1, p < 0.01, permutation test). The other population exhibited increased or decreased responses to both go-cue and no-go-cue odor presentations. Thus, most of the NLOT neurons showed a wide variety of firing rate changes during an odor-sampling epoch.

Based on these response profiles of the odor-sampling epoch, we classified the NLOT neurons into five response types (Figures 2 and S1, see transparent methods). The first neuron group (type I, 38.9% of all neurons) exhibited significant preference for the presented go-cue odor; we will refer to these as “go-cue responsive neurons” (purple pie chart in Figures 2 and S1). The second neuron group (type Ⅱ, 5.8% of all neurons) exhibited significant preference for the presented no-go-cue odor; we will refer to these as “no-go-cue responsive neurons” (orange pie chart in Figures 2 and S1). Two other neuron groups (types III and IV, 11.5% and 17.0% of all neurons, respectively) showed significant excitatory and suppressed responses, respectively, for both presented cue odors without preference for a particular cue odor; we will refer to these as “cue excitatory neurons” (pink pie chart in Figures 2 and S1) and “cue suppressed neurons” (light blue pie chart in Figures 2 and S1), respectively. The remaining neuron group (type Ⅴ, 26.8% of all neurons) did not show significant responses for either presented cue odors; we will refer to these as “cue non-responsive neurons” (gray pie chart in Figures 2 and S1). This classification demonstrated the diverse cue encoding patterns in the NLOT, suggesting that the NLOT neurons did not represent a particular odorant profile from the olfactory bulb; instead, they represented the complex and diverse odor information leading to odor-guided behaviors.

### Go-Cue responsive neurons bidirectionally encode cue odors with excitations and suppressions

**Figure 3:**
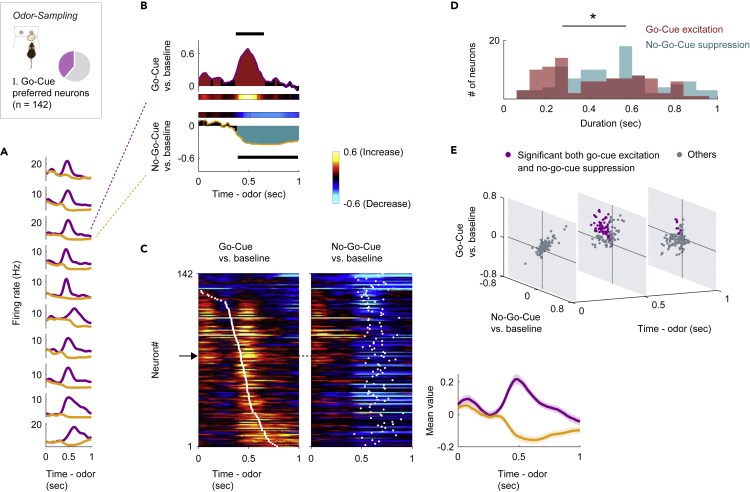
Go-cue responsive neurons show phasic excitation to go-cue odor and persistent suppression to no-go-cue odor (A) Example firing patterns of go-cue responsive neurons during the odor-sampling epoch. Spike histograms are calculated using a 20 ms bin width and smoothed by convolving spike trains with a 60-ms-wide Gaussian filter (purple line, go-cue odor; orange line, no-go-cue odor). (B) Example of the area under the receiver operating characteristic curve (auROC) values for a go-cue responsive neuron. The auROC values (aligned by odor valve opening) were calculated by go-cue odor presentation versus baseline (top), and no-go-cue odor presentation versus baseline (bottom) in the sliding bins (width, 100 ms; step, 20 ms). The red bars show significant excitation, and blue bars show significant suppression (p < 0.01, permutation test). Based on the significant time points, the response “durations” (black horizontal lines) were calculated. (C) The auROC values for go-cue responsive neurons (n = 142, type I neurons). Each row corresponds to one neuron, with neurons in the left and middle graphs in the same order. The neurons are sorted by the times of center of mass (white dots) of the auROC values calculated by go-cue odor presentation versus baseline. The color scale is as in (B). An arrow indicates the same neuron as in (B). The average firing patterns of go-cue responsive group during the odor-sampling epoch (right). (D) Distributions of the response durations for significant excitations (red) and significant suppressions (blue). Statistical significance between excitations and suppressions (∗p < 0.05) was assessed by the Wilcoxon rank-sum test. (E) Time course of excitation to go-cue odor and suppression to no-go-cue odor. Purple dots, significant both go-cue excitation and no-go-cue suppression (p < 0.01, permutation test); gray dots, other responses.

Among the go-cue responsive neurons (type I neurons, n = 142), which represented the major population of the NLOT neurons (Figure 2), each neuron showed a sharp peak in the firing rate after ∼600 ms of go-cue odor presentation and persistent suppression during the latter part of the no-go-cue odor-sampling epoch (Figure 3A). To quantify the dynamics of this bidirectional cue encoding, we calculated the firing rate changes from the baseline (200−0 ms before the end of the inter-trial interval) in the sliding bins during the odor-sampling epoch for each neuron. For each accurate trial type, we calculated the area under the ROC curve (auROC) value at each time bin (width: 100 ms, step: 20 ms) (Figures 3B and 3C) and three measures from the auROC values: “onset time,” “time of center of mass,” and “duration” (see transparent methods). The durations of the go-cue excitation responses were sharper (p < 0.05, Wilcoxon rank-sum test) than those of the no-go-cue suppression responses (Figure 3D). The no-go-cue suppression responses were sustained until the mice withdrew their snouts from the odor port. For each neuron, both the go-cue excitation response and the no-go-cue suppression response were observed at 450–550 ms after the odor onset (Figures 3E and S2, p < 0.01, permutation test). Thus, each go-cue responsive neuron exhibited both sharp go-cue excitation and persistent no-go-cue suppression at specific times during the odor-sampling epoch.

**Figure 4:**
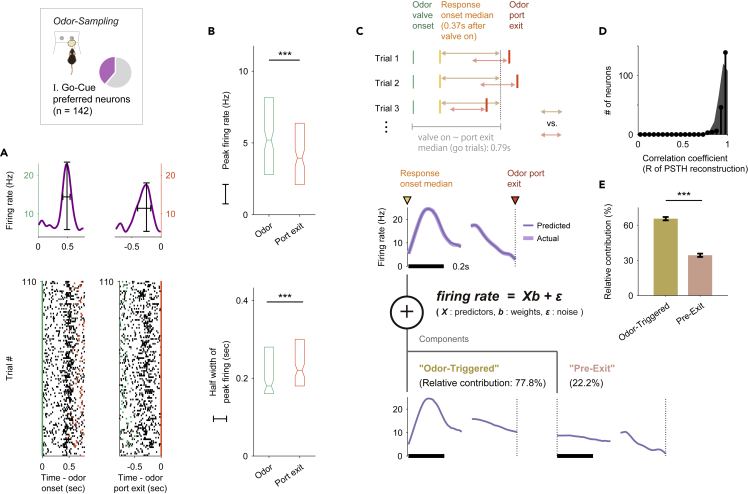
Go-cue excitation response triggered by odor onset rather than the initiation of an odor-guided behavior (A) The activity of an example go-cue responsive neuron aligned to onset of odor valve opening (left, green ticks) or odor port exit (right, red ticks). Raster plots represent the neural activity with each row corresponding to a single trial from the start of the session (bottom) to the 110th trial (top), and each black tick marks a spike. The peak firing rate (black vertical line) and temporal half-width of the peak firing (black horizontal line) are defined from the spike histogram. (B) Comparison of the peak firing rates (top) and half widths of the peak firings (bottom) between the two alignment conditions (odor valve opening versus odor port exit). The peak firing rates were higher when triggered by odor valve opening (∗∗∗p < 10^−15^, Wilcoxon signed-rank test). Half widths of the peak firings were longer when triggered by odor port exit (∗∗∗p < 0.001, Wilcoxon signed-rank test). (C) Schematic of the encoding model used to quantify the relationship between behavioral variables and the activity of each neuron. Inset, predicted and actual averaged firing rate relative to the odor onset and odor port exit for one neuron. (D and E) (D) Correlation coefficients (R of PSTH reconstructions) between predicted and actual averaged firing rate relative to the odor onset and odor port exit across the go-cue responsive neurons. (E) Relative contribution of each behavioral variable to the explained variance of the neural activity, averaged across the go-cue responsive neurons. Relative contribution of the odor-triggered response was significantly higher than that of the pre-odor port exit (∗∗∗p < 10^−9^, Wilcoxon signed-rank test). All error bars are standard error of the mean (SEM).

It is possible that the sharp go-cue excitation responses correlated with the execution of the go behaviors or contained the premotor signals. To verify this possibility, we compared the peak firing rates and the half-width of firing in the go-cue excitation between the two alignment conditions (odor valve opening versus odor port exit). We observed that the peak firing rates were higher relative to the odor onset (p < 10–15, Wilcoxon signed-rank test) and the temporal organizations were significantly tighter (p < 0.001, Wilcoxon signed-rank test) than the firing rate relative to the odor port exit (Figures 4A and 4B). Moreover, we developed an encoding model (generalized linear model) that incorporated task-related variables from 370 ms after the odor onset (corresponding to the time that is the median of the go-cue excitation onsets measured by the auROC values) to the odor port exit as predictors of each neuron's activity to help isolate the go-cue excitation responses triggered by odor onset and action (Figures 4C and 4D) (Engelhard et al., 2019). Using this encoding model, we quantified the relative contribution of each behavioral variable to the response of each neuron by determining how much the explained variance declined when that variable was removed from the model (see transparent methods). Averaged across the go-cue responsive neurons, the relative contribution of the odor-triggered response (65.6% ± 1.4% of the total variance explained, mean ± standard error of mean [SEM]) was significantly higher than that of the pre-odor port exit (34.%4 ± 1.4%) (p < 10^−9^, Wilcoxon signed-rank test) (Figure 4E). These results indicated that the go-cue excitation responses of the go-cue responsive neurons were triggered by odor onset rather than pre-motor activities. Furthermore, distinct cue responses were observed in the correct go trials, and not in the trials that were correct no-go, error, or odorless (Figure S3A), suggesting that the distinct go-cue excitation responses reflected signals eliciting appropriate motivational behavior. Notably, the intensities of the majority of the cue responses remained stable across trials (Figure S3B). In conclusion, the distinct go-cue excitation responses were triggered by odor onset and were stable with respect to the appropriate odor-guided behaviors.

### NLOT neuron population exhibits rapid response dynamics before executing cue-odor-evoked behaviors

We demonstrated that the go-cue responsive neurons exhibited specific temporal dynamics during odor sampling as a representative population of NLOT neurons (Figure 3). Similarly, the no-go-cue responsive neurons exhibited both no-go-cue excitation and persistent go-cue suppression during cue odor presentation (Figures 2 and S5A). The cue excitatory neurons and the cue suppressed neurons also changed their firing rates during odor sampling (Figures 2 and S5A). Thus, the NLOT neurons exhibited diverse firing patterns and complex temporal dynamics during odor sampling. In this section, we examine the NLOT population encoding and the contribution of each neuron group during odor sampling using different methods of analysis.

**Figure 5:**
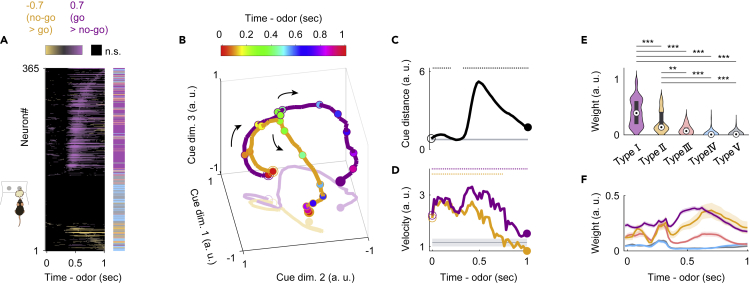
Nucleus of the lateral olfactory tract (NLOT) neuron population response before the initiation of odor-guided behaviors (A) The auROC values (go-cue versus no-go-cue odor presentation, aligned by odor valve opening) for all neurons. Each row corresponds to one neuron. Neurons are sorted by the peak time for the auROC values. The color scale indicates significant preferences (p < 0.01, permutation test; positive values correspond to the go-cue responsive responses). The black boxes indicate bins with non-significant preferences (p > 0.01, permutation test). The colored box on the right shows the neuron type for each neuron (purple, type I; orange, type Ⅱ; pink, type Ⅲ; light blue, type Ⅳ; gray, type Ⅴ). (B) Visualization of the NLOT neuron population responses during odor-sampling epoch using principal-component analysis (n = 365 NLOT neurons). The responses to cue odors are projected onto the first three principal components corresponding to the odor-sampling epoch subspaces. Purple line, go-cue odor; orange line, no-go-cue odor. Temporal progression is depicted from unfilled purple/orange spheres to filled purple/orange spheres. (C) The distance between NLOT neuron population responses. The gray line and shaded areas show the mean ±2 standard deviation (SD) baseline values during the baseline epoch. Top dots indicate the time bins showing values more than mean +2 SD baseline values. (D) Rate of change (velocity) of NLOT neuron population responses. Purple line, go-cue odor; orange line, no-go-cue odor. The gray line and shaded areas show the mean ±2 SD baseline values during the baseline epoch. Top dots indicate the time bins showing values more than mean +2 SD baseline values. (E) Neural weights in the first dimension of the odor-sampling epoch subspaces. Box plots in violin plots indicate medians and interquartile ranges. Purple, type I; orange, type Ⅱ; pink, type Ⅲ; light blue, type Ⅳ; gray, type Ⅴ. The statistical significance among five groups (∗∗p < 0.01, ∗∗∗p < 0.001) was assessed by one-way analysis of variance (ANOVA) with Tukey's post hoc test. (F) Neural weights along the time course in the first dimension of each sliding bin (width: 100 ms, step: 20 ms). The shaded areas represent ±standard error of the mean (SEM). Purple, type I; orange, type Ⅱ; pink, type Ⅲ; light blue, type Ⅳ; gray, type Ⅴ.

Calculating go-cue versus no-go-cue preference during odor sampling clearly showed the strong encodings of cue preference at 400–500 ms after odor onset across the population (Figure 5A, p < 0.01, permutation test). To gain insight into the dynamics of the population response, we visualized the average population activity using principal-component analysis, a dimensionality reduction method (Figure S4A). Figure 5B shows the trajectories of the mean response of the NLOT neuron population to go-cue and no-go-cue odors, represented as the projections onto the first three principal components (PC) during the odor-sampling epoch. Throughout the ∼300-ms interval from the odor onset, trajectories remained converged, showing little difference across conditions. Over the late phase of odor sampling, specifically 400–500 ms from the odor onset, trajectories in the odor-sampling epoch subspace began to spread out and were clearly separated at the population level. To quantify these observations, we measured the instantaneous separation between the population cue responses (Figure 5C). The separation reached a maximum at ∼500 ms and remained above the baseline levels until the odor port exited. Additionally, we calculated the rate at which the population activity vectors changed (width: 100 ms, step: 20 ms; Figure 5D). These rates increased to a maximum within ∼500 ms and remained above the baseline levels over the initiation of cue-odor-evoked behaviors (go or no-go behaviors). Thus the NLOT neuron population showed dramatic transformations in the dynamics of cue encoding at 400–500 ms after odor onset.

Next, we examined the mechanism of the contribution of individual NLOT neurons to the population response to evaluate the absolute values of the PC coefficients as the neural weights (Figures 5E and S4A–S4C). The values of the neural weights in the first dimension of the odor-sampling epoch subspaces showed that type I neurons contributed considerably to the population response. To further examine the contributions along the time course, we calculated the absolute values of the PC coefficients in the sliding bins (width: 100 ms, step: 20 ms) during the odor-sampling epoch (Figures 5F and S4D). The values of the neural weights in the first dimension of each bin exhibited significant contributions of type I neurons to the population response, especially during 400–500 ms after the odor onset, corresponding to the dynamics of cue encoding. These results indicated that the go-cue responsive neurons strongly contributed to the profound transformations in the dynamics of NLOT cue encoding.

### NLOT neurons provided sufficient information to account for behavioral accuracy

**Figure 6:**
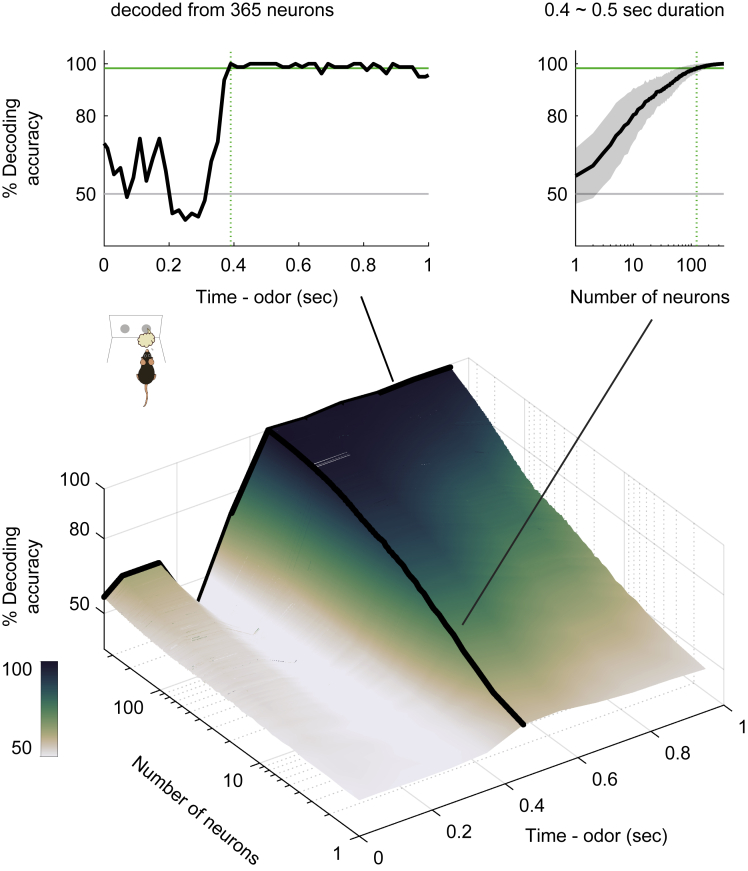
Nucleus of the lateral olfactory tract (NLOT) neurons provided sufficient information to account for behavioral choices Time course of odor decoding accuracy. A vector consisting of instantaneous spike counts for 1–365 neurons in a sliding window (width, 100 ms; step, 20 ms) was used as an input for the classifier. Training of the classifier and testing was performed at every time point. Green horizontal lines indicate the level of animal behavioral performance. Gray horizontal lines indicate chance levels (50%). Green vertical dashed lines indicate the first points wherein the decoding accuracy reached the level of the animal behavioral performance. Areas with shading represent ±SD.

To examine whether the population activity accounted for the animals' behavioral accuracy, we performed a decoding analysis. This analysis determined whether the firing rates of the NLOT neuron populations could be used to classify each individual trial as go or no-go. We used support vector machines with linear kernels as a decoder. Analyses of the decoding time course based on NLOT neurons using a sliding time window revealed that the decoding accuracy was maintained at chance levels 300 ms after odor onset; subsequently, it dramatically increased above the level of animals' behavioral accuracy 400–500 ms after the odor onset (Figure 6). In the 400–500 ms period, 124 neurons provided sufficient information to account for behavioral accuracy (the top right panel in Figure 6). Thus, <150 NLOT neurons provided sufficient information to account for behavioral accuracy at least 500 ms after odor onset.

### Bidirectional cue-outcome encoding following odor-guided behaviors

**Figure 7:**
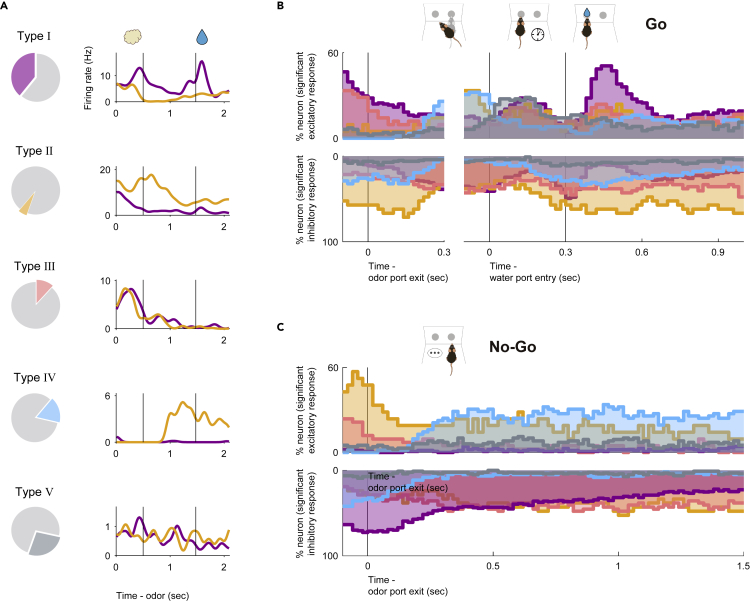
Nucleus of the lateral olfactory tract (NLOT) neurons exhibits bidirectional cue-outcome encoding following odor-guided behaviors (A) Example firing pattern of each neuron group following odor-guided behaviors. Event-aligned spike histograms are calculated using a 20 ms bin width and smoothed by convolving spike trains with a 60-ms-wide Gaussian filter (purple line, go trial; orange line, no-go trial). Vertical black lines indicate the odor valve offset and the onset of water reward. (B and C) (B) The proportions of neurons that exhibited significant responses were calculated from the auROC values (p < 0.01, permutation test) in correct go trials for each neuron group (top, excitation; bottom, suppression). Vertical black lines indicate the odor port exit, water port entry, and the onset of water reward. Purple, type I; orange, type Ⅱ; pink, type Ⅲ; light blue, type Ⅳ; gray, type Ⅴ. (C) Same as (B), for correct no-go trials.

Our analyses of the dynamics of cue encoding suggest that several NLOT neurons maintained cue selective responses during cue-odor-evoked behaviors after odor sampling. Notably, the persistent suppression responses of type I neurons during no-go-cue odor sampling were sustained over the odor port exit (Figures 3B–3D). This raises the question of whether the selectivity disappears or persists once the cue-odor-evoked behaviors are executed. By aligning the neural activity to behavioral events (event-aligned spike histograms, see transparent methods), we noticed that type I neurons were selective for the reward drinking behavior after go-cue odor sampling and the persistent suppression responses were sustained during the no-go waiting behavior after no-go-cue odor sampling (Figure 7A). We quantified the response profiles of each neuron group during odor-evoked behaviors by calculating the firing rate changes from baseline (Figures 7B, 7C, and S5A, the spike data were aligned to the odor port exit and water port entry). Across the population, several type I neurons showed significant excitatory responses for drinking behavior (purple histogram at the top in Figure 7B, p < 0.01, permutation test) and significantly suppressed responses for the no-go waiting behavior (purple histogram at the bottom in Figure 7C, p < 0.01, permutation test). The drinking responses of type I neurons were higher than those of other groups, and the no-go waiting responses of type I neurons were lower than those of other groups, indicating that they were type I neuron-specific responses (Figure S5B, one-way analysis of variance with Tukey's post hoc test). For each neuron, the suppressions were maintained for 800 ms (interquartile range: 290–1,480 ms) from the initiation of the no-go behavior. Thus, type I neurons exhibited associations between the go-cue excitations and excitatory responses for drinking behavior with persistent no-go-cue suppressions, suggesting that NLOT neurons are involved in cue-outcome associations.

We aimed to determine whether other neuron groups responded to cue-odor-evoked behavioral states. Several type II neurons showed significantly suppressed responses for drinking behavior (orange histogram at the bottom in Figure 7B, p < 0.01, permutation test) and significant excitatory responses for the no-go waiting behavior (orange histogram at the top in Figure 7C, p < 0.01, permutation test). The drinking responses of type II neurons were lower than those of type I neurons, and the no-go waiting responses of type II neurons were higher than those of type I neurons (Figure S5B, one-way analysis of variance with Tukey's post hoc test). Thus, the type I and type II neurons contrastingly encoded the go/no-go behavioral states after odor sampling. Furthermore, a subset of types III, IV, and V neurons tended to show an excitatory response in a specific time window in behavioral epochs, with suppressed responses relative to other behavioral epochs (Figure S5C), which is consistent with our previous findings in the ventral tenia tecta of the olfactory cortex (Shiotani et al., 2020). In particular, each type Ⅳ neuron maintained the excitatory response to the no-go waiting state (light blue histogram at the top in Figure 7C, p < 0.01, permutation test) for 560 ms (interquartile range: 205–1,200 ms), from 290 ms (interquartile range: 210–650 ms) after initiation of the no-go behavior. These results indicate that each NLOT neuron group showed a specific firing pattern during odor-guided behaviors, depending on the response profiles in the odor-sampling epochs.

## Discussion

### Electrophysiological features of NLOT neurons

The purpose of the study was to understand the electrophysiological features of NLOT neurons in decision-making processes that occur during various behavioral states in odor-guided go/no-go tasks. In this study, we provided the first recording of neuronal activity in the NLOT in freely behaving mice performing odor-guided go/no-go tasks. Indeed, NLOT neurons exhibited diverse neural activities in response to odor presentations and cue odor-evoked behaviors in the task.

Previous anatomical studies have shown that the NLOT receives odor information from the olfactory bulb and various areas of the olfactory cortex, including the piriform cortex (Luskin and Price, 1983; Price, 1973). Subsequently, NLOT neurons project to the ventral striatum consisting of the olfactory tubercle (OT) and the nucleus accumbens (NAc), and also send the axons into the BLA (Luskin and Price, 1983; Price, 1973; Santiago and Shammah-Lagnado, 2004), which plays a critical role in regulating motivated behaviors (Cox and Witten, 2019; Janak and Tye, 2015; Murata et al., 2015). Moreover, a recent study (Vaz et al., 2017) has shown that NLOT-lesioned rats exhibited olfactory-related behavioral deficits with an inability to identify and discriminate between odors and interfere with the display of innate odor-evoked behaviors, such as sexual behavior, aggression, and avoidance of predators. Despite the accumulation of knowledge, the role of NLOT in the functional circuit to convert odor information into appropriate behaviors has not been clarified.

In this study, we classified five types of neurons based on their firing patterns during the odor-sampling epochs. A majority of NLOT neurons (type I neurons, go-cue responsive neurons) exhibited phasic excitatory responses during go-cue odor-sampling epochs and sustained suppressed responses during no-go-cue odor-sampling epochs (Figures 2, 3, and 4). The activity pattern of the no-go-cue responsive neurons (type II neurons) was opposite to that of the go-cue responsive neurons. These bidirectional cue encoding patterns were similar to the cue encoding in the brain reward circuit, including the ventral striatum and the ventral tegmental area (Cohen et al., 2012; Menegas et al., 2017; Stephenson-Jones et al., 2020) rather than the olfactory circuit (Cury and Uchida, 2010; Miura et al., 2012; Uchida et al., 2014) during cue-outcome association tasks. We also demonstrated that the go-cue and no-go-cue responsive neurons highly contributed to the population dynamics of cue encoding and decoding, for accuracy of the animal's choices (Figure 5), suggesting that these bidirectional response neurons for cue odors effectively provided sufficient information to account for the behavioral choices. These bidirectional cues encoding a small number of neurons with a high level of information may be effective in the NLOT with only a small volume of 0.24 mm^3^ and 19,000 neurons (Vaz et al., 2016).

The go-cue responsive neurons also showed firing activities during drinking behavior (Figure 7B), consistent with other brain areas involved in motivational processes (Steinmetz et al., 2019; Watabe-Uchida et al., 2017). Additionally, the go-cue responsive neurons exhibited suppressed responses to the no-go waiting behavioral states (Figures 7C and S5B). Moreover, the no-go-cue responsive neurons suppressed their firing activity during the go behavioral states and exhibited excitatory activities in the no-go behavioral states (Figures 7B, 7C, and S5B). These results suggest that these NLOT neurons functionally associated cue odor with the precise task outcomes derived from odor information. In other olfactory cortical areas, including the piriform cortex and OT, the odor-reward association reflects the cue odor responses (Calu et al., 2007; Gadziola et al., 2015, 2020; Gadziola and Wesson, 2016; Millman and Murthy, 2020; Roesch et al., 2007). Although further studies are required to determine whether cue responses in the NLOT represent cue-reward learning, these cue-reward combination activities may play a role in odor-reward association learning. We speculated that the NLOT is one of the critical components of the circuitry responsible for creating and providing signals eliciting appropriate behaviors. Owing to the function of NLOT, we assumed that the lesion in NLOT caused suppression of olfactory-driven behaviors (Vaz et al., 2017).

Although we suggested that the distinct go-cue excitation responses of the NLOT neurons reflected motivation signals eliciting appropriate behavior, it may be possible that they reflect action-specific responses, odor-specific responses, and valence-specific responses. We showed that the go-cue excitation responses were triggered by odor onset rather than by action-specific activities (Figure 4). However, we cannot rule out other possibilities because of using one go-cue and one single no-go-cue in this study. Future experiments are required to build on the paradigm to test these possibilities.

The subsets of types III, IV, and V neurons exhibited an excitatory response in a specific time window in behavioral epochs and suppressed responses relative to other behavioral epochs (Figure S5C). Similarly, our recent study has revealed that the individual neuron in the ventral tenia tecta, which is a part of the olfactory cortex, is tuned to a specific behavioral state in mice encountered during odor-guided behaviors (Shiotani et al., 2020). Moreover, a recent study has shown the brain-wide global representation of state-dependent activity during odor-guided motivated behavior (Allen et al., 2019). Together, the context-dependent activities of the types III, IV, and V neurons may contribute to the brain-wide specific information processing mode in the brain, and be shared across the olfactory cortex.

What are the similar and different electrophysiological features between NLOT and other olfactory areas? The olfactory areas, including the olfactory bulb (Cury and Uchida, 2010), showed excitatory responses to some cue-odors during the odor-sampling epoch in a goal-directed task. About 20%–40% of anterior and posterior piriform cortex neurons were activated by at least one of some odors tested in tasks (Calu et al., 2007; Roesch et al., 2007; Miura et al., 2012; Gadziola et al., 2020), whereas about 60%–70% of OT neurons relating to motivational information processing were activated (Gadziola et al., 2015, 2020), corresponding to the NLOT neurons (Figure 2). We noticed that the average peak firing of the cue selective responses in types I and II neurons was later than that of the piriform cortex and OT neurons (Figure 2, Miura et al., 2012). One possibility is that the delayed responses might have been caused by the forced long poke during odor presentation. In this study, the mice were required to keep their nose into the odor port during a 500-ms odor presentation. However, a large number of piriform cortex neurons in rats can discriminate cue odors within 100 ms of the onset of first inhalation during odor presentation in the forced long poke condition (Miura et al., 2012). Indeed, in our experimental conditions, several NLOT and ventral tenia tecta neurons exhibited firing activities on the morrow of the odor valve onset (Figure S5C, Shiotani et al., 2020). Therefore, we assumed that the mice received cue odor information just after odor onset, even in our experimental condition. It is possible that the responses with long latency in the odor presentation state are mainly due to indirect multi-synaptic odor input from the olfactory bulb through the olfactory cortical areas, such as the piriform cortex or top-down inputs from higher brain areas rather than direct mitral cell inputs from the olfactory bulb. If so, as NLOT may receive discriminated odor information, we are also considering one possibility that these delayed responses code for one of the variabilities constituting the building blocks of the decision-making process, which translate odor information into appropriate behavior rather than odor discrimination. Taken together, NLOT may be functionally differentiated from other olfactory cortical areas with respect to odor-guided behavior.

### Neural circuits including the NLOT

Olfactory information is transmitted to the NLOT with a three-layered structure (layers I, II, and III). The NLOT layer II neurons contribute to >80% of the total neuronal population of the NLOT (Vaz et al., 2016) and project to the dwarf cell regions in the OT (Santiago and Shammah-Lagnado, 2004). The OT sends a major projection to the ventral pallidum regulating expected positive and negative valences (Richard et al., 2016; Saga et al., 2017; Tachibana and Hikosaka, 2012). The layer II neurons also project to the NAc shell (Santiago and Shammah-Lagnado, 2004) that processes hedonic or motivational values (Castro et al., 2015; Kelley, 2004; Zorrilla and Koob, 2013). Similar to the neural responses in these areas, we demonstrated the reward-predicting cue and reward signals of type I neurons in the NLOT (Figures 3 and 7). Based on a recent frontal cortex research showing a connectivity-defined neuron type that carries a single variable (Hirokawa et al., 2019), we speculated that type I neuron outputs in the NLOT layer II to the OT and NAc contribute to the encoding of positive or negative valences of expected and actual outcomes, and hedonic or motivational value.

Conversely, the NLOT layer III neurons project to the BLA (Santiago and Shammah-Lagnado, 2004), which is an essential component of the amygdala underlying fear conditioning memory (Janak and Tye, 2015; Zhang et al., 2020). We demonstrated the no-go-cue responses of the type II neurons (Figure 7) and the sustained positive responses to the no-go behaviors of type Ⅳ neurons. We assumed that these specific firing patterns in the NLOT layer III might contribute to the fear-conditioning memory circuits. However, we did not verify the firing pattern of NLOT neurons in fear memory tasks. Future experiments are required to monitor the changes in the firing activity in the NLOT during odor-punishment association tasks.

In conclusion, we extended the concept that NLOT integrity is required for normal functioning of the olfactory system (Vaz et al., 2017), and hypothesized that the NLOT plays a critical role in providing odor information that elicits appropriate behavioral motivation in the motivation circuits in odor-guided behavior. From a broad perspective, the verification of this hypothesis may have important implications for studying and leveraging neural circuits underlying odor-evoked motivation in health and disease.

### Limitation of the study

We acknowledge that there are several limitations to this study. First, although we performed the first *in vivo* recording of neuronal activity in the NLOT during only an odor-guided go/no-go task, our data do not reflect the neuronal activity across different cue modalities, behavioral paradigms, and contexts. However, our data are potentially important in that the NLOT neural activity in freely behaving mice is modulated by the motivation of learned, odor-guided, and goal-directed behaviors and may provide basic information regarding NLOT encoding in positive and negative motivational contexts, reversal learning, and innate odor-driven behaviors (Root et al., 2014). Second, a direct relationship between the distinct cue response of NLOT neurons and context-dependent motivated behaviors is unclear. Third, the response profiles and functions of the NLOT-specific projections to OT, NAc, and BLA on motivational processes have not yet been clarified. By using optogenetic manipulation or the fiber photometry tool to monitor cell-type and projection-specific population activity, future studies can build on the paradigm and findings described here to address how the NLOT interacts with the projected areas to mediate the processes necessary for odor-guided behavior.

### Resource availability

#### Lead contact

Further information and requests for resources and reagents should be directed to and will be fulfilled by the lead contact, Hiroyuki Manabe (hmanabe@mail.doshisha.ac.jp).

#### Materials availability

This study did not generate unique reagent.

#### Data and code availability

The datasets analyzed and produced in the present study are available at https://github.com/Manabe-Team/paper2021_NLOT.

## Methods

All methods can be found in the accompanying Transparent methods supplemental file.

## Supplemental information

Document S1. Transparent methods and Figures S1–S5

## References

[bib1] Alheid G., De Olmos J.S., Beltramino C.A., Paxinos G. (1995). Amygdala and extended amygdala. The Rat Nervous System.

[bib2] Allen W.E., Chen M.Z., Pichamoorthy N., Tien R.H., Pachitariu M., Luo L., Deisseroth K. (2019). Thirst regulates motivated behavior through modulation of brainwide neural population dynamics. Science.

[bib3] Calu D.J., Roesch M.R., Stalnaker T.A., Schoenbaum G. (2007). Associative encoding in posterior piriform cortex during odor discrimination and reversal learning. Cereb. Cortex.

[bib4] Castro D.C., Cole S.L., Berridge K.C. (2015). Lateral hypothalamus, nucleus accumbens, and ventral pallidum roles in eating and hunger: Interactions between homeostatic and reward circuitry. Front. Syst. Neurosci..

[bib5] Cohen J.Y., Haesler S., Vong L., Lowell B.B., Uchida N. (2012). Neuron-type-specific signals for reward and punishment in the ventral tegmental area. Nature.

[bib6] Cox J., Witten I.B. (2019). Striatal circuits for reward learning and decision-making. Nat. Rev. Neurosci..

[bib7] Cury K.M., Uchida N. (2010). Robust odor coding via inhalation-coupled transient activity in the mammalian olfactory bulb. Neuron.

[bib8] Engelhard B., Finkelstein J., Cox J., Fleming W., Jang H.J., Ornelas S., Koay S.A., Thiberge S.Y., Daw N.D., Tank D.W. (2019). Specialized coding of sensory, motor and cognitive variables in VTA dopamine neurons. Nature.

[bib9] Gadziola M.A., Wesson D.W. (2016). The neural representation of goal-directed actions and outcomes in the ventral Striatum’s olfactory tubercle. J. Neurosci..

[bib10] Gadziola M.A., Tylicki K.A., Christian D.L., Wesson D.W. (2015). The olfactory tubercle encodes odor valence in behaving mice. J. Neurosci..

[bib11] Gadziola M.A., Stetzik L.A., Wright K.N., Milton A.J., Arakawa K., Del Mar Cortijo M., Wesson D.W. (2020). A neural system that represents the association of odors with rewarded outcomes and promotes behavioral engagement. Cell Rep..

[bib12] Hirokawa J., Vaughan A., Masset P., Ott T., Kepecs A. (2019). Frontal cortex neuron types categorically encode single decision variables. Nature.

[bib13] Janak P.H., Tye K.M. (2015). From circuits to behaviour in the amygdala. Nature.

[bib14] Kelley A.E. (2004). Ventral striatal control of appetitive motivation: role in ingestive behavior and reward-related learning. Neurosci. Biobehav. Rev..

[bib15] Luskin M.B., Price J.L. (1983). The topographic organization of associational fibers of the olfactory system in the rat, including centrifugal fibers to the olfactory bulb. J. Comp. Neurol..

[bib16] Menegas W., Babayan B.M., Uchida N., Watabe-Uchida M. (2017). Opposite initialization to novel cues in dopamine signaling in ventral and posterior striatum in mice. Elife.

[bib17] Millman D.J., Murthy V.N. (2020). Rapid learning of odor-value association in the olfactory striatum. J. Neurosci..

[bib18] Miura K., Mainen Z.F., Uchida N. (2012). Odor representations in olfactory cortex: distributed rate coding and decorrelated population activity. Neuron.

[bib19] Murata K., Kanno M., Ieki N., Mori K., Yamaguchi M. (2015). Mapping of learned odor-induced motivated behaviors in the mouse olfactory tubercle. J. Neurosci..

[bib20] de Olmos J.S., Beltramino C.A., Alheid G., P.G. (2004). Amygdala and extended amygdala of the rat: a cytoarchitectonical, fibroarchi- tectonical, and chemoarchitectonical survey. The Rat Nervous System.

[bib21] Price J.L. (1973). An autoradiographic study of complementary laminar patterns of termination of afferent fibers to the olfactory cortex. J. Comp. Neurol..

[bib22] Richard J.M., Ambroggi F., Janak P.H., Fields H.L. (2016). Ventral pallidum neurons encode incentive value and promote cue-elicited instrumental actions. Neuron.

[bib23] Roesch M.R., Stalnaker T.A., Schoenbaum G. (2007). Associative encoding in anterior piriform cortex versus orbitofrontal cortex during odor discrimination and reversal learning. Cereb. Cortex.

[bib24] Root C.M., Denny C.A., Hen R., Axel R. (2014). The participation of cortical amygdala in innate, odour-driven behaviour. Nature.

[bib25] Saga Y., Richard A., Sgambato-Faure V., Hoshi E., Tobler P.N., Tremblay L. (2017). Ventral pallidum encodes contextual information and controls aversive behaviors. Cereb. Cortex.

[bib26] Santiago A.C., Shammah-Lagnado S.J. (2004). Efferent, connections of the nucleus of the lateral olfactory tract in the rat. J. Comp. Neurol..

[bib27] Shiotani K., Tanisumi Y., Murata K., Hirokawa J., Sakurai Y., Manabe H. (2020). Tuning of olfactory cortex ventral tenia tecta neurons to distinct task elements of goal-directed behavior. Elife.

[bib28] Steinmetz N.A., Zatka-Haas P., Carandini M., Harris K.D. (2019). Distributed coding of choice, action and engagement across the mouse brain. Nature.

[bib29] Stephenson-Jones M., Bravo-Rivera C., Ahrens S., Furlan A., Xiao X., Fernandes-Henriques C., Li B. (2020). Opposing contributions of GABAergic and glutamatergic ventral pallidal neurons to motivational behaviors. Neuron.

[bib30] Swanson L.W., Petrovich G.D. (1998). What is the amygdala?. Trends Neurosci..

[bib31] Tachibana Y., Hikosaka O. (2012). The primate ventral pallidum encodes expected reward value and regulates motor action. Neuron.

[bib32] Uchida N., Poo C., Haddad R. (2014). Coding and transformations in the olfactory system. Annu. Rev. Neurosci..

[bib33] Vaz R.P., Pereira P.A., Madeira M.D. (2016). Age effects on the nucleus of the lateral olfactory tract of the rat. J. Comp. Neurol..

[bib34] Vaz R.P., Cardoso A., Sá S.I., Pereira P.A., Madeira M.D. (2017). The integrity of the nucleus of the lateral olfactory tract is essential for the normal functioning of the olfactory system. Brain Struct. Funct..

[bib35] Watabe-Uchida M., Eshel N., Uchida N. (2017). Neural circuitry of reward prediction error. Annu. Rev. Neurosci..

[bib36] Zhang X., Kim J., Tonegawa S. (2020). Amygdala reward neurons form and store fear extinction memory. Neuron.

[bib37] Zorrilla E.P., Koob G.F. (2013). Amygdalostriatal projections in the neurocircuitry for motivation: a neuroanatomical thread through the career of Ann Kelley. Neurosci. Biobehav. Rev..

