# Supplementary material for: Bi-directional encoding of context-based odors and behavioral states by the nucleus of the lateral olfactory tract
Source: iScience. 2021 Mar 31;24(4):102381. doi: 10.1016/j.isci.2021.102381 (PMC8082085; doi:10.1016/j.isci.2021.102381)
Supplement: Document S1. Transparent methods and Figures S1–S5 [file mmc1.pdf]

iScience, Volume *24*

Supplemental information

Bi-directional encoding of context-based odors and behavioral states by the nucleus of the lateral olfactory tract

Yuta Tanisumi, Kazuki Shiotani, Junya Hirokawa, Yoshio Sakurai, and Hiroyuki Manabe

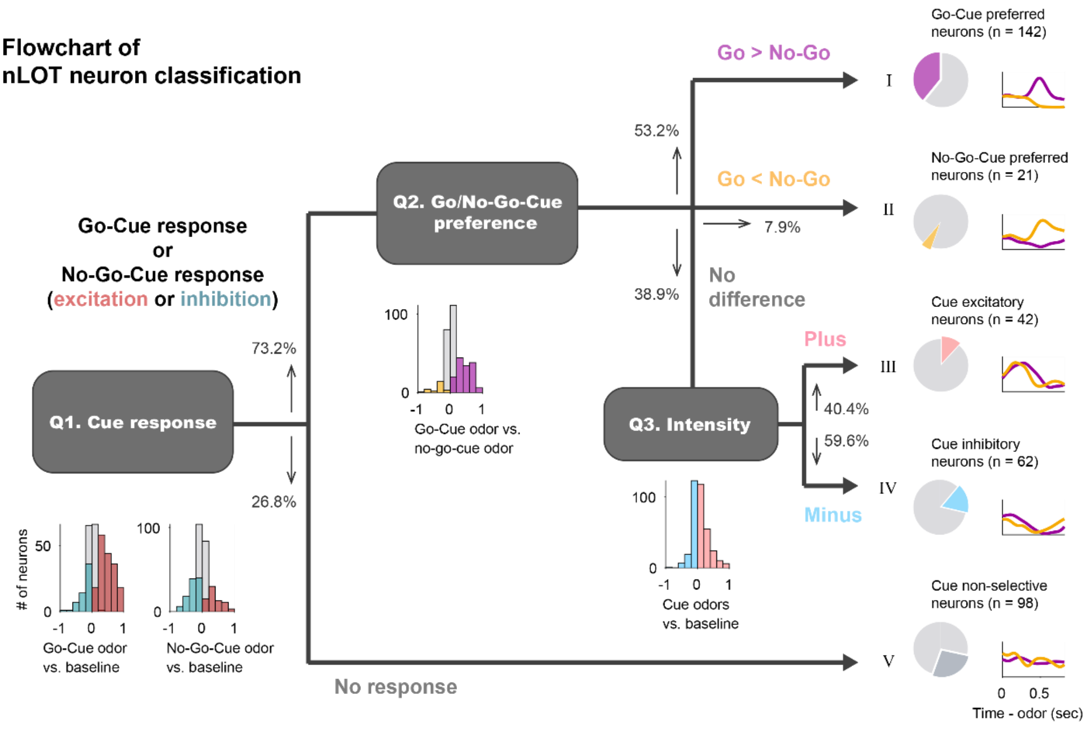

Figure S1. Flowchart of Nucleus of the Lateral Olfactory Tract (NLOT) Neuron Classification, related to Figure 2.
We classified the NLOT neurons into five types based on the response profiles of the odor-sampling epoch. First (Q1), we calculated the area under the receiver operating characteristic curve (auROC) values of go-cue versus baseline and no-go-cue versus baseline during the odor-sampling epoch in the correct trials (red histogram, significant excitation; blue histogram, significant suppression). Based on these values, we defined the cue odor selective population (73.2%) that exhibited significant responses in at least one cue odor presentation and cue odor non-selective population (26.8%, type V neurons). Second (Q2), in the cue odor selective population, we also calculated the auROC values of go-cue versus no-go-cue during the odor-sampling epoch in the correct trials (purple histogram, significant go-cue > no-go-cue; orange histogram, significant go-cue < no-go-cue). Based on these values, we defined go-cue responsive neurons (53.2%, type I neurons) and no-go-cue responsive neurons (7.9%, type II neurons). Finally (Q3), in the remaining population (38.9%), we calculated the auROC values of cue odors (go-cue + no-go-cue) versus baseline during the odor-sampling epoch in the correct trials (pink histogram, excitation; light blue histogram, suppression). Based on these values, we defined cue excitatory neurons (40.4%, type III neurons) and cue suppressed neurons (59.6%, type IV neurons). For all aforementioned analyses, we tested for significance at $\alpha = 0.01$ (permutation test).

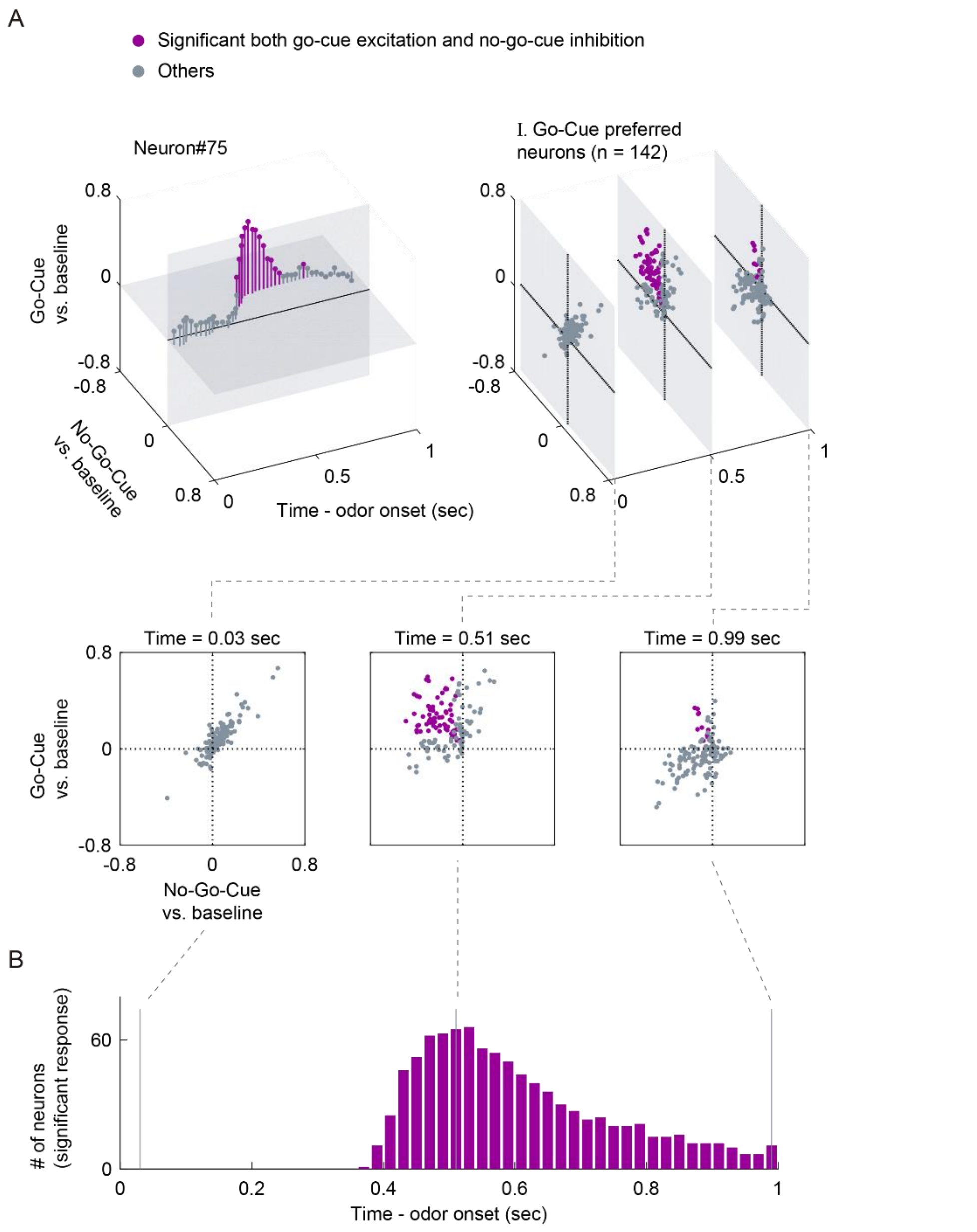

Figure S2. Evaluation of Go-Cue Excitation and No-Go-Cue Suppression Responses, related to Figure 3.

(**A**) Time course of excitation to go-cue odor and suppression to no-go-cue odor. Purple dots, significant both go-cue excitation and no-go-cue suppression ($p < 0.01$, permutation test); gray dots, other responses.

(**B**) The number of neurons that exhibited significant responses calculated from the auROC values ($p < 0.01$, permutation test).

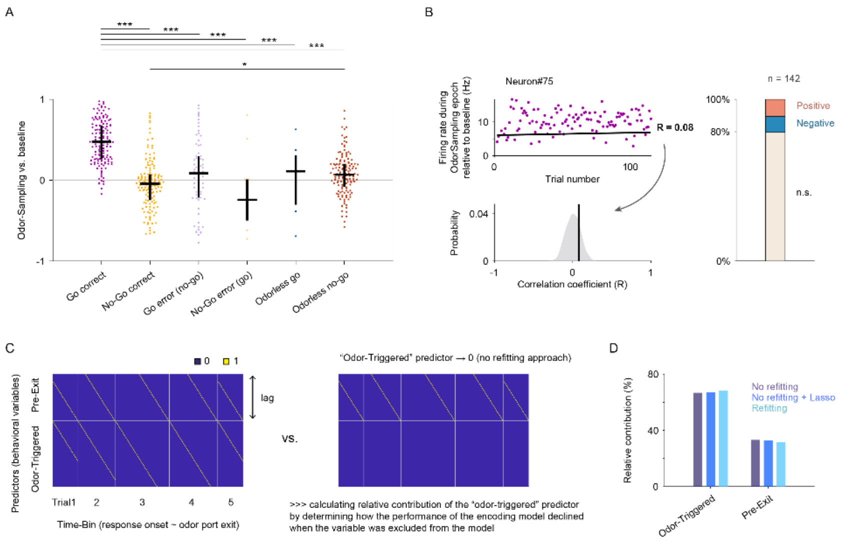

Figure S3. Go-Cue Excitation Responses Reflected Signals Eliciting Appropriate Motivational Behavior and Stable, related to Figure 4.

(**A**) Go-cue excitation and no-go-cue suppression responses during correct trials, error trials, and catch (odorless) trials. The area under the receiver operating characteristic curve (auROC) values were calculated during the odor-sampling epochs and only neurons with a minimum number of three trials for each analyzed condition were included in this analysis. Black horizontal lines and black vertical lines indicate medians and interquartile ranges. The statistical significance among six groups (*$p < 0.05$, ***$p < 0.001$) was assessed by one-way analysis of variance (ANOVA) with Tukey's post hoc test.

(**B**) The development of cue responses in go-cue responsive neurons during learning. For each go-cue responsive neuron, we calculated the correlation between the firing rate during the go-cue odor-sampling epoch relative to the baseline (a mean firing rate during inter trial interval was subtracted for each neuron) and the order of go trial from the start of the session. The correlation coefficient was compared with control values calculated using the 1000 trial-shuffled data (gray shaded area) and the statistical significance was determined (<0.5th percentiles of the control values, negative correlation; >99.5th percentiles of the control values, positive correlation). Across go-cue responsive neurons, the majority of the go-cue responses were not correlated with trial progression (79.5%, not significant; 9.9%, negative; 10.6%, positive).

(**C**) Structure of predictor matrices. The predictor has rows for each variable and time offset, which take non-zero values for time points (columns) corresponding to the appropriate time offset from the given event. We quantified the relative contribution of each behavioral variable to the response of each neuron by determining how much the explained variance declined when that variable was removed from the model.

(**D**) Average relative contributions across the go-cue responsive neurons assessed separately using three different approaches: no refitting (used in the paper); no refitting + Lasso regularization; and refitting. Lasso regularization was applied using the lasso function in MATLAB; the mean square error (MSE) of the model was estimated using fivefold cross-validation, and we chose the lambda value that minimized the MSE. The results with lasso regularization were almost identical to the result without regularization, which suggests that there was not significant overfitting in our model.

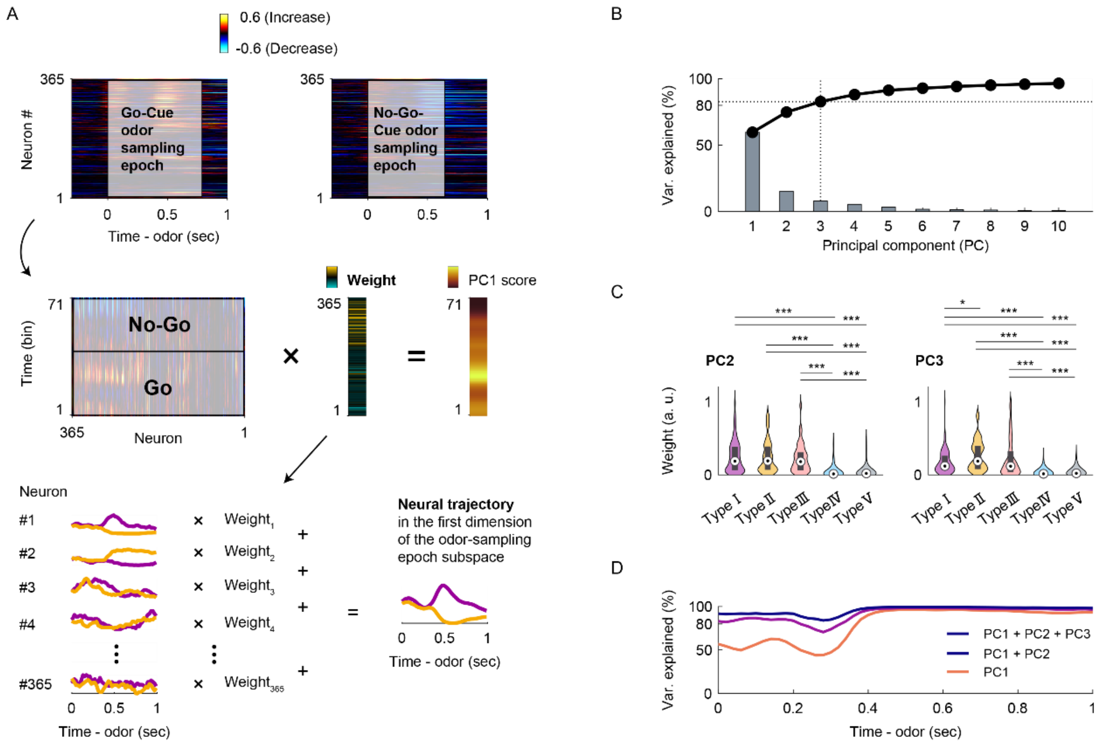

Figure S4. Population Vector Construction and Analyses for the Nucleus of the Lateral Olfactory Tract (NLOT) Neuron Population Response, related to Figure 5.
(**A**) Population vector construction. We constructed the two conditions (71 time bins) × 365 neurons matrix during the odor-sampling epoch, in which the columns contained the auROC values corresponding to the trial-averaged firing rate changes from the baseline. By performing principal component analysis (PCA) on the data, we reduced the dimensionality of the NLOT population from 365 neurons to three principal components (PCs). Subsequently, we obtained the odor-sampling epoch subspaces and neural weights (graphs show the values of the first dimension of the odor-sampling epoch subspaces).
(**B**) Screen plot of the odor-sampling epoch subspaces. It is notable that we used the three subspaces as they explained 82.8% of the total variance.
(**C**) Neural weights in the second (left) and third (right) dimension of the odor-sampling epoch subspaces. Box-plots in violin-plots indicate the medians and interquartile ranges. Purple, type I; orange, type II; pink, type III; light blue, type IV; gray, type V. Statistical significance among five groups (*$p < 0.05$, ***$p < 0.001$) was assessed by one-way analysis of variance (ANOVA) with Tukey's post hoc test.
(**D**) Variances of neural weights data along the time course (**Figure 5F**) in the dimensions of each sliding bin (width: 100 ms, step: 20 ms).

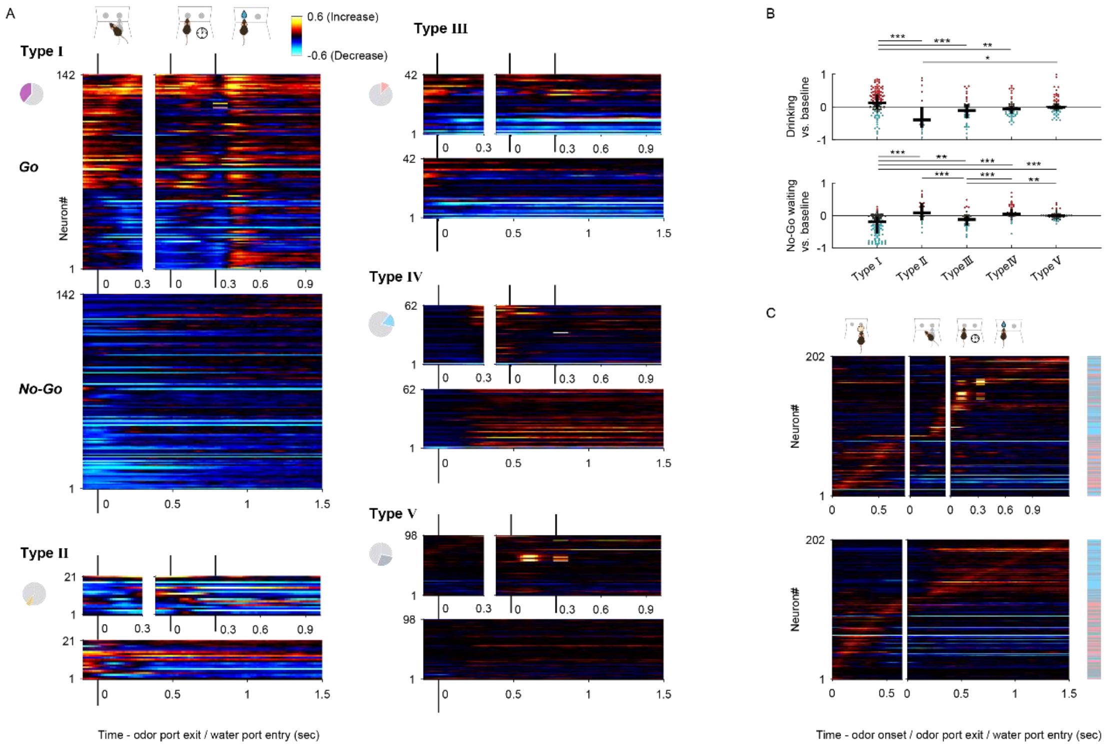

Figure S5. Response Profiles Following Odor-Guided Behaviors, related to Figure 7.

(**A**) The area under the receiver operating characteristic curve (auROC) values calculated by go or no-go trials versus the baseline in the sliding bins (width, 100 ms; step, 20 ms) following odor-guided behaviors. Each row corresponds to one neuron, with neurons in all the graphs in the same order for each neuron group. Neurons were sorted by the peak time for the auROC values. The color scale is as in **Figure 3C**.

(**B**) The auROC values during the drinking epoch (top) and no-go waiting epoch (bottom). Black horizontal lines and black vertical lines indicate the medians and interquartile ranges. Red dots, significant excitation; blue dots, significant suppression; gray dots, non-significant ($p < 0.01$, permutation test). Statistical significance among five groups (*$p < 0.05$, **$p < 0.01$, ***$p < 0.001$) was assessed by one-way analysis of variance (ANOVA) with Tukey's post hoc test.

(**C**) The auROC values calculated by go or no-go trials versus the baseline in the sliding bins (width, 100 ms; step, 20 ms) during odor-guided go/no-go task in the type III, IV, and V neurons. Each row corresponds to one neuron. Neurons are sorted by the peak time for the auROC values. The color scale is as in **Figure 3C**. The colored box on the right shows neuron type for each neuron (pink, type III; light blue, type IV; gray, type V). Note that these neurons tended to show an excitatory response to a specific behavioral epoch with suppressed responses relative to other behavioral epochs.

Transparent Methods

Animals

All the experiments were performed on male C57BL/6 mice (9 weeks old; weighing 20–25 g), purchased from Shimizu Laboratory Supplies Co., Ltd., Kyoto, Japan. The mice were individually housed in a temperature-controlled environment with a 13-hr light/11-hr h dark cycle (lights on at 8:00 and off at 21:00). They were provided with water after the training and recording sessions to ensure that the body weights dipped no lower than 85% of the initial levels, and food was supplied ad libitum. All experiments were performed in accordance with the guidelines for animal experiments at Doshisha University and with the approval of the Doshisha University Animal Research Committee.

Apparatus

We used a behavioral apparatus controlled by the Bpod State Machine r0.5 (Sanworks LLC, NY), an open-source control device designed for behavioral tasks. The apparatus comprised a custom-designed mouse behavior box with two nose-poke ports on the front wall. The box was contained in another soundproof box (BrainScience Idea. Co., Ltd., Osaka, Japan) equipped with a ventilator fan that provided adequate air circulation and low-level background noise. Each of the two nose-poke ports had a white light-emitting diode (LED) and infrared photodiode. Interruption of the infrared beam generated a transistor-transistor-logic (TTL) pulse; thus, signaling the entry of the mouse head into the port. The odor delivery port was equipped with stainless steel tubing connected to a custom-made olfactometer (Uchida and Mainen, 2003). Eugenol was used as the go-cue odor and amyl acetate (Tokyo Chemical Industry Co., Ltd., Tokyo, Japan) as the no-go-cue odor. These odors were diluted to 10% in mineral oil and further diluted to 1:9 by airflow. Water-reward delivery was based on gravitational flow, controlled by a solenoid valve (The Lee Company, CT), and connected via Tygon tubing to the stainless steel tubing. The reward amount (6 μL) was determined by the opening duration of the solenoid valve and was regularly calibrated.

Odor-Guided go/no-go task

After a 3 s inter-trial interval, each trial began by illuminating the LED light at the right odor port, which instructed the mouse to poke its nose into that port. A nose-poke into the odor port resulted in the delivery of one of the two cue odors for 500 ms. The mice were required to maintain their nose-poke during odor stimulation to sniff the odor. After odor stimulation, the LED light was turned off and the mice could withdraw their noses from the odor ports. If a eugenol odor (go-cue odor) was presented, the mice were required to move to and nose-poke into the left water reward port within a timeout period of 2 s. At the water port, the mice were required to maintain their nose-poke for 300 ms before water delivery began. Next, 6 μL of water was delivered as a reward. If an amyl acetate odor (no-go-cue odor) was presented, the mice were required to avoid entering the water port for 2 s following odor stimulation. Once every 10 trials, we introduced catch trials in which the airstream was delivered through a filter containing no odorants during which, the mice were not rewarded regardless of their choice (go or no-go behavior). During the training sessions, the mice learned to obtain water rewards at the left water port, move from the right odor port to the left odor port, and associate odor cues with the correct

action. The accuracy rate was calculated as the total percentage of successes in the go and no-go trials in a session. The mice performed up to 448 trials (go error: ~20 trials, no-go error: ~4 trials, go in catch trials: ~11 trials, no-go in catch trials: ~37 trials) in each session per day.

To test the time lag between the start of odor stimulation and the arrival of the odor molecules to the mouse's nose, we conducted additional experiments in which odor presentation durations were 100, 200, 300, and 500 ms, without keeping the mice's nose inserted into the odor port during the odor presentation in each session (14, 6, 11, and 7 sessions in three mice). The odor presentation duration was fixed in each session and reduced from 500 ms to 100 ms across sessions.

Electrophysiology

The mice were anesthetized with medetomidine (0.75 mg/kg i.p.), midazolam (4.0 mg/kg i.p.), and butorphanol (5.0 mg/kg i.p.), and implanted with a custom-built microdrive of four tetrodes in the NLOT (0.1 mm anterior to the bregma, 2.0 mm lateral to the midline). Individual tetrodes consisted of four twisted polyimide-coated tungsten wires (California Fine Wire, single wire diameter 12.5 μm, gold plated to <500 kΩ). Two additional screws were threaded into the bone above the cerebellum for reference. The electrodes were connected to an electrode interface board (EIB-18, Neuralynx, MT) on the microdrive. The microdrive array was fixed to the skull using LOCTITE 454 (Henkel Corporation, Düsseldorf, Germany). After the completion of surgery, the mice received atipamezole (0.75 mg/kg i.p.) to reverse the effects of medetomidine and allow for a shorter recovery period. Additionally, the mice received analgesics (ketoprofen, 5 mg/kg, i.p.). Behavioral training resumed at least 1 week postoperatively. Electrical signals were obtained using open-source hardware (Open Ephys). For unit recordings, the signals were sampled at 30 kHz in Open Ephys and band-pass filtered at 600–6,000 Hz. After each recording, the tetrodes were adjusted to obtain new units.

Data analyses

All data analyses were performed using built-in and custom-built software in MATLAB 2019a (The Mathworks, Inc., MA).

Spike sorting: The spikes were sorted into clusters offline based on their waveform energy, peak amplitudes, and the first principal components from the four tetrode channels using an automated spike-separation algorithm KlustaKwik (K.D. Harris). The resulting classifications were corrected and refined manually using MClust software (A.D. Redish). The clusters were considered as single units only when the following criteria were met: (1) refractory period (2 ms) violations were <0.2% of all spikes, and (2) the isolation distance, estimated as the distance from the center of the identified cluster to the nearest cluster based on the Mahalanobis distance, was >20.

Spike train analyses: Neural and behavioral data were synchronized by inputting each event timestamp from the Bpod behavioral control system into the electric signal recording system. To calculate the firing rates during tasks, peri-event time histograms (PETHs) were calculated using a 20 ms bin width and smoothed by convolving spike trains with a 60 ms-wide Gaussian filter.

To examine the relationship between the firing rate changes among individual NLOT neurons and the development of behavioral epochs in behavioral tasks, we created event-aligned spike histograms (EASHs) (Ito and Doya, 2015). As behavioral epoch durations varied for each trial, the median duration of the epoch was calculated first. In the odor-guided go/no-go task, the median duration of odor-sampling epochs (from the odor onset to the odor port exit) was 788 ms in the go trials, 642 ms in the no-go trials, and the median duration of moving epochs (from the odor port exit to the water port entry) was 388 ms. The spike timing during each epoch and for each trial was linearly transformed to correspond with the median behavioral duration of each epoch. The number of spikes in each epoch was preserved. Furthermore, we defined the waiting epoch (300 ms reward delay, from the water port entry to the onset of water reward) and the drinking epoch (1,000 ms after the onset of water reward). These epochs were not applied to the transformation as their durations did not change across trials. In this way, the regular raster plots were transformed into event-aligned raster plots. Consequently, an EASH was calculated using a 20 ms bin width and smoothed by convolving the spike trains with a 60 ms-wide Gaussian filter from the event-aligned raster plots (**Figure 7A**).

ROC analyses: To quantify the firing rate changes, we used an algorithm based on ROC analyses that calculates the ability of an ideal observer to classify whether a given spike rate was recorded in one of two conditions (e.g., during go-cue or no-go-cue odor presentation) (Felsen and Mainen, 2008). We defined an auROC equal to 2 (ROCarea - 0.5), with the measure ranging from -1 to 1, where -1 signifies the strongest possible value for one alternative and 1 signifies the strongest possible value for the other.

The statistical significance of these ROC analyses was determined using a permutation test. For this test, we recalculated the ROC curves after randomly reassigning all firing rates to either of the two groups arbitrarily. This procedure was repeated a large number of times (500 repeats for analyses of dynamics [**Figures 3D-E, S2, 5A, and 7B-C**], 1,000 repeats for all other analyses; we shuffled only the trial labels) to obtain a distribution of values. Subsequently, we calculated the fraction of random values exceeding the actual value. For all analyses, we tested for significance at $\alpha = 0.01$. Only neurons (sessions) with a minimum number of three trials for each analyzed condition were included in the analyses.

For analyses of dynamics (width: 100 ms, step: 20 ms), we calculated three measures from the auROC values of correct trials (**Figures 3D and 7B-C**):

(1) Time of center of mass: This refers to the time corresponding to the center of mass of the significant points of the auROC values ($p < 0.01$, permutation test). The center of mass was calculated as the average of the histogram ($\sum_i[\text{class value}_i \text{ frequency}_i] / \sum_i \text{frequency}_i$). Only neurons with significant points for each analyzed condition were included in this analysis.

(2) Duration: This refers to the duration in which the auROC values were significant ($p < 0.01$, permutation test) for ≥5 consecutive bins, containing the time of center of mass. Only neurons with consecutive bins for each analyzed condition were included in this analysis.

(3) Onset time: The onset time refers to the time at which the duration was first evident.

Classification of NLOT neurons: Based on the ROC analyses during the odor-sampling epoch, we classified the NLOT neurons into five types (**Figures 2 and S1**). First, we

calculated the auROC values of the go-cue versus baseline (1,000 to 0 ms before the end of the inter-trial interval) and the no-go-cue versus baseline during the odor-sampling epoch in the correct trials. Based on these values, we defined the cue odor selective population that exhibited significant responses for at least one cue odor presentation and cue odor non-selective population (type V neurons). Second, in the cue odor selective population, we also calculated the auROC values of the go-cue versus the no-go-cue during the odor-sampling epoch in the correct trials. Based on these values, we defined go-cue responsive neurons (significant go-cue > no-go-cue, type I neurons) and no-go-cue responsive neurons (significant go-cue < no-go-cue, type II neurons). Finally, in the remaining population, we also calculated the auROC values of cue odors (go-cue + no-go-cue) versus baseline during the odor-sampling epoch in the correct trials. Based on these values, we defined cue excitatory neurons (cue odors > baseline, type III neurons) and cue suppressed neurons (cue odors < baseline, type IV neurons). For all aforementioned analyses, we tested for significance at $\alpha = 0.01$ (permutation test).

Generalized linear models: To quantify the contribution of behavioral variables to neural activity, we used generalized linear models (GLM), which was a multiple linear regression with the firing rate of each neuron as the dependent variable, and predictors derived from the behavioral variables as the independent variables (**Figures 4C-D and S3C**) (Engelhard et al., 2019). In this analysis, the firing rate (5 ms bin width and smoothed by convolving spike trains with a 25 ms-wide Gaussian filter) of each neuron is described as a linear sum of temporal filters aligned to task events. For this study, only the onset of go-cue excitation response and pre-odor port exit events in correct go trials were required, since we consider only the period in between them (370 ms after odor stimulus onset to odor port exit). In the model, the predicted firing rate is given as:

$$\widehat{y_t} = \beta_0 + \sum_i \beta_i^{Odor-Triggered} x_{t-i}^{Odor-Triggered} + \sum_i \beta_i^{Pre-Exit} x_{t-i}^{Pre-Exit}$$

The response of a neuron at bin t is modeled ($\widehat{y_t}$) by the sum of a bias term (β_0) and the weighted (β_i) sum of various additional binary predictors at different lags (i). Binary predictors for the odor-triggered response ($x_t^{Odor-triggered}$) are supported over the window of 370–788 ms relative to the onset of odor valve in either correct go trials (lags corresponding to the period from the onset of go-cue excitation response to the median of the odor port exit, 84 time bins). Binary predictors for pre-odor port exit events ($x_t^{Pre-Exit}$) are supported over the window of -300–0 ms relative to the odor port exit in either go or no-go trials (60 time bins). The β values were calculated using the glmfit MATLAB function.

Calculation of the relative contributions of behavioral variables to neural activity: We quantified the relative contribution of each behavioral variable to neural activity (**Figures 4E and S3C**) by determining how the performance of the encoding model declined when each variable was excluded from the model (Engelhard et al., 2019; Pho et al., 2018). We predicted the firing rate of each neuron with all variables (full model) or by excluding one of the variables (partial model). The relative contribution of each behavioral variable was calculated by comparing the variance explained of the partial model to the variance explained by the full model. For this study, which included two behavioral variables, the relative contribution of each variable was defined as

$$\left(1-\frac{R_{p,i}^2}{R_f^2}\right)/\sum_{j=1}^{2}\left(1-\frac{R_{p,j}^2}{R_f^2}\right)$$

Here, $R_{p,i}^2$ is the variance explained by the partial model that excludes the *i*th variable, and R_f^2 is that of the full model. Negative relative contributions were set to zero (this occurs when the R^2 of the full model is lower than that of the partial model, owing to the introduction of noise by the excluded variable).

We used two approaches to exclude variables from the full model and calculate the variance explained by the partial model (Engelhard et al., 2019). In the first approach, the partial model was equivalent to the full model, except that the β values of the predictors of the excluded variable were set to zero (‘no refitting’). In the second approach, we calculated new β values by re-running the regression without the predictors of the excluded variable (refitting). Both approaches to exclude variables produced comparable results; the no-refitting approach was used to generate the main figures, and comparison with the refitting approach is shown in **Figure S3D**.

Moreover, we compared relative contributions assessed separately using three different approaches: no refitting (NR; used in the paper), no refitting + Lasso regularization (NR + L), and refitting (R). Lasso regularization was applied using the lasso function in MATLAB; the mean square error (MSE) of the model was estimated using fivefold cross-validation, and we chose the lambda value that minimized the MSE. The results with lasso regularization were almost identical to the result without regularization (**Figure S3D**), which suggests that there was no significant overfitting in our model.

Population vector construction and analyses: We constructed two conditions (71 time bins) × 365 neurons matrix (Cavanagh et al., 2018; Murray et al., 2017; Ohnuki et al., 2020) during the odor-sampling epoch, in which columns contained the auROC values of the correct trials corresponding to the trial-averaged firing rate changes from the baseline (**Figure S4A**). By performing principal component analysis (PCA) on the data, we reduced the dimensionality of the NLOT population from 365 neurons to three PCs and obtained the odor-sampling epoch subspaces. Notably, we used the three subspaces as 82.8% of the total variance was explained (**Figure S4B**). To visualize the NLOT population responses, we projected the data onto the three-dimensional subspaces (**Figure 5B**). This allowed us to obtain a point reflecting the response of the entire population for each of the two conditions at a given instant. The distance between the cue responses was computed as the Euclidean distance between pairs of activity vectors of all subspaces at a given instant (**Figure 5C**) (Cury and Uchida, 2010; Mazor and Laurent, 2005). The velocity of population responses was determined as the distance between successive 20 ms bins (**Figure 5D**) (Mazor and Laurent, 2005). These values were compared with the values during the baseline epoch (200–0 ms before the end of the inter-trial interval).

To examine the contribution of individual neurons to cue encoding, we evaluated the absolute values of the PC coefficients as the neural weights (**Figures 5E and S4C**). We also evaluated contributions along the time course by calculating the absolute values of the PC coefficients in the sliding bins (width: 100 ms, step: 20 ms) during odor-sampling (**Figures 5F and S4D**).

SVM decoding analyses: We used a SVM algorithm with a linear kernel as a classifier (Cury and Uchida, 2010; Miura et al., 2012) and a MATLAB function (fitcsvm) for analyses. All analyses were conducted on trial data pooled across animals. A matrix containing concatenated firing rates for each trial and each neuron provided input to the classifier. The matrix dimensions were the number of cells by the number of trials. To avoid over-fitting, k-fold cross-validation (k = 10) was used to calculate the decoding accuracy of trial type discrimination. To compute the decoding accuracy, 40 trials for each trial type (from the start of the session) were chosen as the data. Next, the data were partitioned into ten equal parts; one part was used for testing and the remaining parts were used for training the classifier. This process was repeated ten times to test each individual part; the mean value of the accuracy was used for decoding accuracy. To compute the decoding accuracy of a 100 ms bin window (step: 20 ms), the classifier was trained and tested with a 100 ms bin window (step: 20 ms).

Statistical analyses: The data were analyzed using MATLAB 2019a. The statistical methods used in each analysis have been described in the Results section or figure legends. The Tukey-Kramer method was applied for tests of significance with multiple comparisons. Although the sample sizes in this study were not pre-determined by sample size calculations, they were based on previous research in the olfactory cortex fields (Manabe et al., 2011; Miura et al., 2012). Randomization and blinding were not employed. Biological replicates for the histological studies are described in the figure legends.

Histology

After recording, the mice were deeply anesthetized using an intraperitoneal injection of sodium pentobarbital. Electric lesions were made using 10–20 μA direct current stimulation for 5 s of one of the four tetrode leads. The mice were perfused transcardially with phosphate-buffered saline (PBS) and subsequently with 4% paraformaldehyde. The brains were removed from the skull and post-fixed in PFA. Next, the brains were cut into 50-μm thick coronal sections and stained with cresyl violet. The electrode track positions were determined in reference to the atlas developed by Paxinos and Watson (Paxinos, 2004).

Supplemental References

Cavanagh, S.E., Towers, J.P., Wallis, J.D., Hunt, L.T., and Kennerley, S.W. (2018). Reconciling persistent and dynamic hypotheses of working memory coding in prefrontal cortex. Nat. Commun. 9, 1–16.

Cury, K.M., and Uchida, N. (2010). Robust odor coding via inhalation-coupled transient activity in the mammalian olfactory bulb. Neuron 68, 570–585.

Engelhard, B., Finkelstein, J., Cox, J., Fleming, W., Jang, H.J., Ornelas, S., Koay, S.A., Thiberge, S.Y., Daw, N.D., Tank, D.W., et al. (2019). Specialized coding of sensory, motor and cognitive variables in VTA dopamine neurons. Nature 570, 509–513.

Felsen, G., and Mainen, Z.F. (2008). Neural Substrates of Sensory-Guided Locomotor Decisions in the Rat Superior Colliculus. Neuron 60, 137–148.

Ito, M., and Doya, K. (2015). Distinct neural representation in the dorsolateral, dorsomedial, and ventral parts of the striatum during fixed- and free-choice tasks. J. Neurosci. 35, 3499–3514.

Manabe, H., Kusumoto-Yoshida, I., Ota, M., and Mori, K. (2011). Olfactory cortex generates synchronized top-down inputs to the olfactory bulb during slow-wave sleep. J. Neurosci. 31, 8123–8133.

Mazor, O., and Laurent, G. (2005). Transient dynamics versus fixed points in odor representations by locust antennal lobe projection neurons. Neuron 48, 661–673.

Millman, D.J., and Murthy, V.N. (2020). Rapid Learning of Odor-Value Association in the Olfactory Striatum. J. Neurosci. 40, 4335–4347.

Miura, K., Mainen, Z.F., and Uchida, N. (2012). Odor representations in olfactory cortex: distributed rate coding and decorrelated population activity. Neuron 74, 1087–1098.

Murray, J.D., Bernacchia, A., Roy, N.A., Constantinidis, C., Romo, R., and Wang, X.J. (2017). Stable population coding for working memory coexists with heterogeneous neural dynamics in prefrontal cortex. Proc. Natl. Acad. Sci. U. S. A. 114, 394–399.

Ohnuki, T., Osako, Y., Manabe, H., Sakurai, Y., and Hirokawa, J. (2020). Dynamic coordination of the perirhinal cortical neurons supports coherent representations between task epochs. Commun. Biol. 3, 406.

Paxinos, G. (2004). The mouse brain in stereotaxic coordinates / George Paxinos, Keith Franklin (London: Academic).

Pho, G.N., Goard, M.J., Woodson, J., Crawford, B., and Sur, M. (2018). Task-dependent representations of stimulus and choice in mouse parietal cortex. Nat. Commun. 9, 2596.

Uchida, N., and Mainen, Z.F. (2003). Speed and accuracy of olfactory discrimination in the rat. Nat Neurosci 6, 1224–1229.
